# CVOT summit report 2024: new cardiovascular, kidney, and metabolic outcomes

**DOI:** 10.1186/s12933-025-02700-0

**Published:** 2025-05-02

**Authors:** Oliver Schnell, Jaime Almandoz, Lisa Anderson, Katharine Barnard-Kelly, Tadej Battelino, Matthias Blüher, Luca Busetto, Doina Catrinou, Antonio Ceriello, Xavier Cos, Thomas Danne, Colin M. Dayan, Stefano Del Prato, Beatriz Fernández-Fernández, Paola Fioretto, Thomas Forst, James R. Gavin, Francesco Giorgino, Per-Henrik Groop, Igor A. Harsch, Hiddo J. L. Heerspink, Lutz Heinemann, Mahmoud Ibrahim, Michel Jadoul, Sarah Jarvis, Linong Ji, Naresh Kanumilli, Mikhail Kosiborod, Ulf Landmesser, Sofia Macieira, Boris Mankovsky, Nikolaus Marx, Chantal Mathieu, Barbara McGowan, Tatjana Milenkovic, Othmar Moser, Dirk Müller-Wieland, Nikolaos Papanas, Dipesh C. Patel, Andreas F. H. Pfeiffer, Dario Rahelić, Helena W. Rodbard, Lars Rydén, Elke Schaeffner, C. Wendy Spearman, Alin Stirban, Frank Tacke, Pinar Topsever, Luc Van Gaal, Eberhard Standl

**Affiliations:** 1 Forschergruppe Diabetes e. V., Helmholtz Center Munich, Ingolstaedter Landstraße 1, 85764 Neuherberg (Munich), Germany; 2 Division of Endocrinology, Department of Internal Medicine, University of Texas Southwestern Medical Center, Dallas, TX USA; 3 Molecular and Clinical Sciences Research Institute, St. George’s University of London, London, UK; 4 St. George’s University Hospitals NHS Foundation Trust, London, UK; 5 Southern Health NHS Foundation Trust, Southampton, UK; 6 University Medical Center, Ljubljana, Slovenia; 7 Faculty of Medicine, University of Ljubljana, Ljubljana, Slovenia; 8 Helmholtz Institute for Metabolic, Obesity and Vascular Research (HI-MAG) of the Helmholtz Zentrum München at the University of Leipzig and University Hospital Leipzig, Leipzig, Germany; 9 Medical Department III-Endocrinology, Nephrology, Rheumatology, University of Leipzig Medical Center, Leipzig, Germany; 10 Department of Medicine (DIMED), University of Padova, Padua, Italy; 11 Faculty of Medicine, Ovidius University of Constanta, Constanta, Romania; 12 IRCCS MultiMedica, Milan, Italy; 13 DAP Cat Research Group, Foundation University Institute for Primary Health Care Research Jordi Gol i Gorina, Barcelona, Spain; 14 NOVA Medical School, Lisbon, Portugal; 15 Cardiff University School of Medicine, Cardiff, UK; 16 Interdisciplinary Research Center “Health Science”, Sant’Anna School of Advanced Studies, Pisa, Italy; 17 Division of Nephrology and Hypertension, University Hospital Fundación Jiménez Díaz, Madrid, Spain; 18 Faculty of Medicine, Autonomous University of Madrid, Madrid, Spain; 19 Faculty of Medicine, University of Padova, Padua, Italy; 20 CRS Clinical Research Services Mannheim GmbH, Mannheim, Germany; 21 Emory University School of Medicine, Atlanta, GA USA; 22 Department of Precision and Regenerative Medicine and Ionian Area, University of Bari Aldo Moro, Bari, Italy; 23 Department of Nephrology, University of Helsinki and Helsinki University Hospital, Helsinki, Finland; 24 Folkhälsan Research Center, Biomedicum, Helsinki, Finland; 25 Department of Diabetes, Central Medical School, Monash University, Melbourne, Australia; 26 Baker Heart and Diabetes Institute, Melbourne, VIC Australia; 27 Division of Endocrinology and Metabolism, Department of Internal Medicine II, Thuringia Clinic Saalfeld “Georgius Agricola”, Saalfeld, Germany; 28 Department of Clinical Pharmacy and Pharmacology, University Medical Centre Groningen, University of Groningen, Groningen, The Netherlands; 29 Science Consulting in Diabetes GmbH, Dusseldorf, Germany; 30 EDC, Centre for Diabetes Education, Charlotte, USA; 31 Division of Nephrology, Cliniques Universitaires Saint-Luc, Université Catholique de Louvain, Brussels, Belgium; 32 General Practitioner, London, UK; 33 Peking University People’s Hospital, Xicheng District, Beijing, China; 34 Manchester University Foundation Trust, Manchester, UK; 35 Department of Cardiovascular Disease, Saint Luke’s Mid America Heart Institute, University of Missouri-Kansas City School of Medicine, Kansas City, MO USA; 36 Department of Cardiology Angiology and Intensive Care Medicine, Charité Universitätsmedizin Berlin, Berlin, Germany; 37 Sciarc GmbH, Baierbrunn, Germany; 38 Depatment of Diabetology, Shupyk National Healthcare University of Ukraine, Kiev, Ukraine; 39 Clinic for Cardiology, Pneumology, Angiology and Internal Intensive Care Medicine (Medical Clinic I), RWTH Aachen University Hospital, Aachen, Germany; 40 Department of Endocrinology, Catholic University of Louvain, Louvain, Belgium; 41 Guy’s and St Thomas’ Hospital, Kings College London, London, UK; 42 University Clinic of Endocrinology, Diabetes and Metabolic Diseases, Skopje, North Macedonia; 43 Faculty of Medicine “St. Cyril and Methodius” University, Skopje, North Macedonia; 44 Institute of Sports Science, University of Bayreuth, Bayreuth, Germany; 45 Interdisciplinary Metabolic Medicine Trials Unit, Division of Endocrinology and Diabetology, Medical University of Graz, Graz, Austria; 46 Department of Medicine I, University Hospital Aachen, Aachen, Germany; 47 Diabetes Centre-Diabetic Foot Clinic, Second Department of Internal Medicine, Democritus University of Thrace, Alexandroupolis, Greece; 48 Royal Free London, University College London, London, UK; 49 Department of Endocrinology and Metabolic Diseases, Charité Universitätsmedizin Berlin, Campus Benjamin Franklin, Berlin, Germany; 50 Deutsches Zentrum für Diabetesforschung e.V., Helmholtz Center Munich, Neuherberg, Germany; 51 Vuk Vrhovac University Clinic for Diabetes, Endocrinology and Metabolic Diseases at Merkur University Hospital, Zagreb, Croatia; 52 Endocrine and Metabolic Consultants, Rockville, MD USA; 53 Department of Medicine K2, Karolinska Institute, Stockholm, Sweden; 54 Institute of Public Health, Charité Universitätsmedizin Berlin, Berlin, Germany; 55 Division of Hepatology, Department of Medicine, Faculty of Health Sciences, University of Cape Town, Cape Town, South Africa; 56 Asklepios Klinik Birkenwerder, Birkenwerder, Germany; 57 Department of Hepatology and Gastroenterology, Charité - Universitätsmedizin Berlin, Campus Virchow-Klinikum and Campus Charité Mitte, Berlin, Germany; 58 Department of Family Medicine, Acıbadem Mehmet Ali Aydınlar University School of Medicine, Istanbul, Turkey; 59 Department of Endocrinology-Diabetology and Metabolism, Antwerp University Hospital, Antwerp, Belgium

**Keywords:** Cardiovascular disease, Chronic kidney disease, CGM, CKM, Diabetes, Finerenone, GLP-1 RA, Guidelines, Heart failure, MASLD, Obesity, SGLT2 inhibitor, Tirzepatide

## Abstract

The 10th Cardiovascular Outcome Trial (CVOT) Summit: Congress on Cardiovascular, Kidney, and Metabolic Outcomes was held virtually on December 5–6, 2024. This year, discussions about cardiovascular (CV) and kidney outcome trials centered on the recent findings from studies involving empagliflozin (EMPACT-MI), semaglutide (STEP-HFpEF-DM and FLOW), tirzepatide (SURMOUNT-OSA and SUMMIT), and finerenone (FINEARTS-HF). These studies represent significant advances in reducing the risk of major adverse cardiovascular events (MACE) and improving metabolic outcomes in heart failure with preserved ejection fraction (HFpEF), chronic kidney disease (CKD), and obstructive sleep apnea (OSA). The congress also comprised sessions on novel and established therapies for managing HFpEF, CKD, and obesity; guidelines for managing CKD and metabolic dysfunction-associated steatotic liver disease (MASLD); organ crosstalk and the development of cardio-kidney-metabolic (CKM) syndrome; precision medicine and person-centered management of diabetes, obesity, cardiovascular disease (CVD) and CKD; early detection of type 1 diabetes (T1D) and strategies to delay its onset; continuous glucose monitoring (CGM) and automated insulin delivery (AID); cardiovascular autonomic neuropathy (CAN) and the diabetic heart; and the role of primary care in the early detection, prevention and management of CKM diseases. The contribution of environmental plastic pollution to CVD risk, the increasing understanding of the efficacy and safety of incretin therapies in the treatment of CKM diseases, and the latest updates on nutrition strategies for CKM management under incretin-based therapies were also topics of interest for a vast audience of endocrinologists, diabetologists, cardiologists, nephrologists and primary care physicians, who actively engaged in online discussions. The 11th CVOT Summit will be held virtually on November 20–21, 2025 (http://www.cvot.org).

## Background

Since its inception 10 years ago, the Cardiovascular Outcome Trial (CVOT) Summit has been held annually to provide the medical community with the latest knowledge and evidence from cardiovascular (CV), kidney, and metabolic outcome trials and their translation into clinical practice. CVOTs became mandatory in 2008, when the U.S. Food and Drug Administration (FDA) issued a guidance to industry [1], which is currently being updated [2], requiring that all new type 2 diabetes (T2D) therapies be evaluated in long-term CVOTs. Since then, new classes of glucose-lowering drugs have been introduced for the treatment of T2D, namely di-peptidyl peptidase-4 inhibitors (DPP-4is), sodium-glucose cotransporter-2 inhibitors (SGLT2is), glucagon-like peptide 1 receptor agonists (GLP-1 RAs) and, since 2022, tirzepatide, a dual glucose-dependent insulinotropic polypeptide (GIP)/ GLP-1 RA. Finerenone, a nonsteroidal mineralocorticoid receptor antagonist (nsMRA), has also been studied and approved for patients with T2D and chronic kidney disease (CKD).

The prevalence of diabetes in adults worldwide has surpassed 800 million, according to new data [3]. Another source of concern, linked to the increasing prevalence of diabetes, is the increasing prevalence of obesity. Nearly 880 million adults and 159 million children and adolescents are currently living with this condition [4].

Most people with T2D live with overweight or obesity [5], and for each unit increase in body mass index (BMI), the likelihood of diabetes increases significantly [6]. Moreover, both T2D and obesity are independently associated with an increased risk of CV complications and diseases, which are the cause of death for at least half of individuals with T2D [7] and more than two thirds of individuals with high BMI [8]. Further major complications of T2D and obesity are CKD [9–11], (heart failure (HF) [12], metabolic dysfunction-associated steatotic liver disease (MASLD) [13] and obstructive sleep apnea (OSA) [14, 15].

Several clinical studies have demonstrated a cause-and-effect relationship between obesity and T2D, unraveling their strong connections, and it is well established that effective management of obesity can delay the progression of prediabetes to T2D and improve glycemic control in diabetes [16]. Conversely, studies with GLP-1 RAs and dual GIP/GLP-1 RAs, originally developed as glucose lowering drugs, have shown that these substances are very effective in reducing body weight [17]. Therefore, not only CVOTs, but also HF, kidney and metabolic outcomes trials have been conducted with the new T2D medications.

By 2023, five CVOTs each for DPP-4is [18–22] and SGLT2is [23–27], and seven CVOTs with GLP-1 RAs [28–34] have been published. Regarding finerenone, one CVOT [35] was completed. Six HF trials were performed with SGLT2is [36–41] and one with GLP-1 RA [42]. Three kidney outcome trials have been completed with SGLT2is [43–45] and one with finerenone [46].

In general, CVOTs with DPP-4is, GLP-1RAs, SGLT2is, and finerenone included a three-point composite endpoint of major adverse cardiovascular events (3P-MACE): CV death, nonfatal myocardial infarction (MI), and nonfatal stroke. DPP-4is were noninferior to placebo in the 3P-MACE [18–22]. However, saxagliptin, among DPP-4is, was associated with an increased risk of hospitalization for heart failure (HHF) [18]. In addition, CV benefits, including reduction in the number of HHF, have been observed with SGLT2is [23–27, 36–40], some GLP-1 RAs [28–33], and finerenone [35, 46, 47].

In metabolic outcome trials, significant effects on weight reduction were shown with semaglutide (GLP-1RA) [48–51] and tirzepatide (dual GIP/GLP-1 RA)[52–57] in people with or without T2D. Recently, the 3-year safety outcomes of the SURMOUNT-1 trial with tirzepatide (dual GIP/GLP-1 RA) were reported, confirming its efficacy in reducing weight and delaying progression to T2D in persons with both obesity and prediabetes [58]. Results of the SURMOUNT-5 trial (ClinicalTrials.gov Identifier:NCT05822830), which evaluated the efficacy and safety of tirzepatide (dual GIP/GLP-1 RA) compared with semaglutide (GLP-1 RA) in adults with obesity or overweight, at least one weight-related comorbidity but no T2D, are in process and expected to be presented and published in 2025. Recently reported top-line results indicate a 47% greater relative weight loss with tirzepatide compared to semaglutide over 72 weeks of treatment [59].

In 2024, the outcomes of four further HF outcome trials with empagliflozin (EMPACT-MI) [60], semaglutide (STEP-HFpEF-DM)[61], tirzepatide (SUMMIT)[62], and finerenone (FINEARTS-HF) [63] were published, as well as a kidney outcome trial with semaglutide (FLOW) [64], and a metabolic outcome trial with tirzepatide (SURMOUNT-OSA)[65]. Two further CVOTs are currently being conducted with tirzepatide: SURPASS-CVOT (ClinicalTrials.gov Identifier: NCT04255433) in people with T2D and a history of cardiovascular disease (CVD) and SURMOUNT-MMO (ClinicalTrials.gov Identifier: NCT05556512) in people living with overweight or obesity. The efficacy and safety of tirzepatide in CKD is being evaluated in the TREASURE-CKD (ClinicalTrials.gov Identifier: NCT05536804) trial.

Following the practice of previous years [66–74], we present and summarize the key aspects discussed at the 10th CVOT Summit: Congress on Cardiovascular, Kidney, and Metabolic Outcomes held virtually on December 5–6, 2024. The Summit was an interdisciplinary platform organized in collaboration with four study groups: Primary Care Diabetes Europe (PCDE, www.pcdeurope.org), Diabetes and Cardiovascular Disease Study Group (DCVD, www.dcvd.org), Forschergruppe Diabetes e.V., Munich, Germany, and the Working Group “Diabetes and the Heart” of the German Diabetes Society (DDG) (www.ddg.org), and endorsed by four scientific societies: European Association for the Study of Obesity (www.easo.org), European Renal Association (www.era-online.org), China CardioMetabolic Association, and Diabetes India (www.diabetesindia.com).

## Updates on cardiovascular outcome trials (CVOTs)

A summary of the characteristics and results of HF, kidney, and metabolic outcome trials with SGLT2is, GLP-1 RAs, dual GIP/GLP-1 RAs, and nsMRAs published in 2024 is listed in Tables1, 2, 3, 4, 5 and 6.

### Sodium-glucose cotransporter-2 (SGLT2) inhibitors

#### EMPACT-MI [60]

The event-driven EMPACT-MI trial investigated the effect of empagliflozin (10 mg/daily) added to standard of care in 6522 patients who had been hospitalized with an acute MI within 14 days before randomization and had either evidence of a new onset left ventricular ejection fraction (LEVF) of less than 45% or signs or symptoms of congestion leading to treatment during the index hospitalization (or both) [60]. Participants were eligible if they were ≥ 18 years old and had at least one additional clinical risk factor known to be associated with HHF or death from any cause. Such clinical factors included an age of 65 years or older; a newly developed LVEF < 35%; history of MI, atrial fibrillation, or T2D; an estimated glomerular filtration rate (eGFR) of less than 60 ml/min/1.73 m^2^ of body surface area; an elevated level of natriuretic peptide or uric acid; an elevated pulmonary artery or right ventricular systolic pressure; three-vessel coronary artery disease; peripheral artery disease; or no revascularization for the index MI. Exclusion criteria included a previous diagnosis of HF and current or planned treatment with an SGLT2 inhibitor [60].

Patients were randomized 1:1 to receive empagliflozin (n = 3260) or placebo (n = 3262) and were followed for a median of 17.9 months. The primary endpoint was defined as a composite of HHF or death from any cause as assessed in a time-to-first-event analysis. A prespecified hierarchical testing procedure was used, beginning with the primary endpoint and then proceeding to the set of key secondary endpoints, which included the total number of HHF or death from any cause, the total number of nonelective CV hospitalizations or death from any cause, the total number of nonelective hospitalizations for any cause or death from any cause, and the total number of hospitalizations for MI or death from any cause (Table 1) [60].

**Table 1:** Key information of the EMPACT-MI trial [60]

EMPACT-MI [60]	
Class & cardiovascular (CV) outcomes	Effect of empagliflozin vs placebo (95% CI)*
*Primary endpoint*	
Composite of first HHF and death from any cause	0.90 (0.76 to 1.06)^§¥^
*Key secondary endpoints*	
Total no. of HHF or death from any cause	0.87 (0.68 to 1.10)^#^
Total no. of nonelective CV hospitalizations or death from any cause	0.92 (0.78 to 1.07)^#^
Total no. of nonelective hospitalizations for any cause or death from any causes	0.87 (0.77 to 1.0)^#^
Total no. of hospitalizations for MI or death from any cause	1.06 (0.83 to 1.35)^#^
*Other secondary endpoints of interest*	
CV death	1.03 (0.81 to 1.31)^§^
Total no. of HHF	0.67 (0.5 to 0.89)^#^

*Effect presented as HR or as RR.

^§^HR estimated with the use of Cox proportional hazard models.

¥p = 0.21.

^#^RR estimated with the use of negative binomial regression analysis.

*CI* Confidence interval, *CV* Cardiovascular, *HF* Heart failure, *HHF* Hospitalization for HF, *HR* Hazard ratio, *MI* Myocardial infarction, *RR* Rate ratio.

The primary composite endpoint and its components were analyzed according to the intention-to-treat principle and the between-group differences in the risk of a primary endpoint event were assessed with the use of a Cox proportional-hazards model that included the baseline covariates of age, geographic region, eGFR, LVEF, T2D status, atrial fibrillation, previous MI, peripheral artery disease, and smoking status. The key secondary endpoints were analyzed with a negative binomial regression model [75] using the same covariates as for the primary endpoint and the logarithm of time as an adjustment for observation time.

The primary composite endpoint event occurred in 267 patients (8.2%) in the empagliflozin group and in 298 patients (9.1%) in the placebo group, with incidence rates of 5.9 and 6.6 events, respectively, per 100 patient-years (hazard ratio [HR], 0.90; 95% confidence interval [CI], 0.76 to 1.06; p = 0.21) (Table 1) [60]. Since EMPACT-MI did not meet its primary endpoint, secondary and further analyses described below should be interpreted as exploratory. A first HHF occurred in 118 (3.6%) patients in the empagliflozin group and in 153 (4.7%) patients in the placebo group (HR 0.77; 95% CI 0.60 to 0.98), and death from any cause occurred in 169 (5.2%) and 178 (5.5%) patients, respectively (HR 0.96; 95% CI 0.78 to 1.19) [60]. There were 317 (9.7%) and 385 (11.8%) cases of HHF or death from any cause in the empagliflozin and placebo groups, respectively [60]. Nonelective CV hospitalization or death from any cause occurred in 666 (20.4%) patients in the empagliflozin group and 730 (22.3%) patients in the control group, while 998 (30.6%) and 1138 (34.9%) patients in the empagliflozin and placebo groups, respectively, had non-elective CV hospitalization or death from any causes [60]. Similar numbers of hospitalizations for MI or death from any cause were observed in both groups [60]. The rate ratio (RR) (empagliflozin vs. placebo) for each of these key secondary endpoints is shown in Table 1. In addition, CV death occurred in 132 (4.0%) patients in the empagliflozin group and 131 (4.0%) patients in the placebo group (HR 1.03; 95% CI 0.81 to 1.31). The total number of HHF events was 148 (4.5%) in the empagliflozin group and 207 (6.3%) in the placebo group, with rates of 2.4 and 3.6 events per 100 patient-years, respectively, (RR, 0.67; 95% CI 0.51 to 0.89) (Table 1) [60]. The percentage of serious adverse events was similar in the treatment and control groups [60].

### Glucagon-like peptide 1 (GLP-1) receptor agonists

#### STEP-HFpEF DM [61].

The STEP-HFpEF trial [61] evaluated the safety and efficacy of semaglutide (2.4 mg/weekly) injected subcutaneously in 616 patients with HFpEF, obesity and T2D. Participants ≥ 18 years of age were eligible if they had a LVEF ≥ 45%, a BMI ≥ 30, a diagnosis of T2D at least 90 days prior to screening, and a glycated hemoglobin (HbA1c) level no greater than 10% (86 mmol/mol) [61]. At least one of the following findings was also required: elevated left ventricular filling pressures; elevated natriuretic peptide levels plus echocardiographic abnormalities; or HHF within 12 months before screening plus echocardiographic abnormalities or ongoing treatment with diuretics [61]. Key exclusion criteria were a change in body weight of more than 5 kg within 90 days before screening, a history of type 1 diabetes (T1D), use of a GLP-1 RA within 90 days before screening, and uncontrolled diabetic retinopathy [61].

Randomization of eligible individuals was stratified according to BMI (< 35 vs. ≥ 35 kg/m^2^). Participants were then randomly assigned in a 1:1 ratio to receive either semaglutide (n = 310) or placebo (n = 306) for 52 weeks followed by a 5-week follow-up period [61]. The target dose of 2.4 mg of semaglutide was achieved gradually after 16 weeks of treatment, with the starting dose of semaglutide being 0.25 mg once weekly for 4 weeks [61].

Two primary endpoints were specified: a change in the Kansas City Cardiomyopathy Questionnaire clinical summary score (KCCQ-CSS) and the percentage change in body weight from baseline to week 52 (Table 2) [61]. Three confirmatory secondary endpoints were defined: the 6-min walk distance from baseline to week 52, a hierarchical composite endpoint, and a change in the log-transformed C-reactive protein (CRP) level from screening (week -2) to week 52 (Table 2) [61]. The hierarchical composite endpoint included death from any cause from baseline to week 57, the number and timing of HF events requiring hospitalization or urgent HF visit from baseline to week 57, differences of at least 15, 10, or 5 points in KCCQ-CSS change between baseline and week 52 and a difference of at least 30 m in the change in the 6-min walk distance from baseline to week 52 [61].

**Table 2:** Key information of the STEP-HFpEF DM trial [61]

STEP-HFpEF DM [61]		
Class & cardiovascular (CV) outcomes	Estimated difference or ratio (95% CI)	p-value
*Dual primary endpoints*		
Change in KCCQ-CSS from baseline to week 52 (points)	7.3 (4.1 to 10.4)*	< 0.001
Change in body weight from baseline to week 52 (%)	-6.4 (-7.6 to -5.2)*	< 0.001
*Confirmatory secondary endpoints*		
Change from baseline to week 52 in 6-min walk distance (m)	14.3 (3.7 to 24.9)*	0.008
Hierarchical composite endpoint (crude % of wins)	1.58 (1.29 to 1.94)^§^	< 0.001
Change from baseline to week 52 in CRP level (%)	0.67 (0.55 to 0.80)^#^	< 0.001

Adverse events	Event rate (%) active vs. placebo group	p-value
*Serious adverse events*	17.7 vs 28.8	0.002
Cardiac disorders	6.1 vs 13.1	0.004
Gastrointestinal disorders	1.6 vs 1.6	1.0
*Adjudicated events*		
Death from any cause	1.9 vs 3.3	–
CV death	0.3 vs. 1.3	–
HF event	2.3 vs. 5.9	–

*Estimated between-group difference.

§Odds-ratio.

#Estimated treatment ratio (i.e., the ratio [semaglutide:placebo] between the geometric mean ratios of the week 52 value to the baseline value). The ratio to baseline and the corresponding baseline value were log-transformed before analysis. The approximate relative changes were derived from estimated ratios by subtracting 1 and multiplying by 100. The geometric mean ratio of the week 52 value to the baseline value was 0.58 in the semaglutide group and 0.87 in the placebo group. The estimated treatment ratio is calculated as 0.58/0.87 = 0.67.

*CI* Confidence interval, *CV* Cardiovascular, *HF* Heart failure, *KCCQ-CSS* Kansas City Cardiomyopathy Questionnaire clinical summary score.

Two estimands were used to evaluate treatment efficacy: a treatment policy estimand (akin to an intention-to-treat analysis) and a hypothetical trial product estimand (if treatment was taken as intended, or an on-treatment analysis) [61]. The treatment policy estimand assesses the treatment effect regardless of whether treatment is discontinued, or a rescue intervention is received, and it was used to assess the results of the dual primary and the confirmatory secondary endpoints shown in Table 2. Continuous endpoints at week 52 in this trial (the dual primary and the secondary endpoints) were evaluated using analysis of covariance [61]. The hierarchical composite endpoint was evaluated using the win ratio approach [76, 61]. The corresponding value presented in Table 2 is an odds ratio. The CRP levels in the two groups were compared by first estimating the geometric mean ratio of the week 52 value to the baseline value for each group, and then calculating the ratio between the values obtained for the semaglutide and placebo groups [61].

Analysis of the dual primary endpoints showed a mean change in the KCCQ-CSS at week 52 of 13.7 points in the semaglutide group and 6.4 points in the placebo group (estimated difference, 7.3 points; 95% CI 4.1 to 10.4; p < 0.001) (Table 2) [61], and a significant reduction in body weight at week 52 of 9.8% in the semaglutide group and 3.4% in the placebo group (estimated difference in mean body weight, − 6.4; 95% CI, − 7.6 to − 5.2; p < 0.001) (Table 2) [61]. Results of the confirmatory secondary endpoints showed a mean change in the 6-min walk distance at week 52 of 12.7 m in the semaglutide group and -1.6 m in the placebo group (estimated difference 14.3 m; 95% CI 3.7 to 24.9; p = 0.008) (Table 2) [61], and greater efficacy for semaglutide than for placebo in the hierarchical composite secondary endpoint with a difference of at least 15 points in the change in KCCQ-CSS contributing the most wins for semaglutide [61]. The stratified win ratio was 1.58 (95% CI 1.51 to 1.94; p < 0.001) (Table 2) [61].

Participants in the semaglutide group had a 42.0% reduction in CRP level at 52 weeks (geometric mean ratio [week 52 value to baseline value], 0.58), as compared with a 12.8% reduction with placebo (geometric mean ratio [week 52 value to baseline value], 0.87) (estimated treatment ratio 0.67; 95% CI 0.55 to 0.80; p < 0.001) (Table 2) [61]. Before log transformation, the estimated CRP levels at week 52 were 4.44 mg/l in the semaglutide group and 6.08 mg/l in the placebo group, and the changes in estimated CRP levels from baseline to week 52 were–2.48 mg/l and–0.84 mg/l, respectively [61].

Serious adverse events were reported in 17.7% (n = 55) of the participants in the semaglutide group and 28.8% (n = 88) participants in the placebo group (p = 0.002) (Table 2) and were mainly attributed to cardiac disorders [61], which were reported in 6.1% (n = 19) of the patients in the semaglutide group and in 13.1% (n = 40) of the patients in the placebo group (p = 0.004) (Table 2). Gastrointestinal disorders were reported by 5 (1.6%) patients in each group(p = 1.00) (Table 2) [61].

Death from any cause, death from CV causes, and HF events were adjudicated as adverse events by an external committee [61]. A total of 16 patients died during the study, 6 in the semaglutide group and 10 in the placebo group, for an incidence of 1.9% and 3.3%, respectively (Table 2) [61]. Five deaths were attributed to CV causes (1 in the semaglutide group and 4 in the placebo group) [61]. Seven patients in the semaglutide group and 18 patients in the placebo group experienced an HF event (HHF or urgent visit for HF) [61].

#### FLOW [64]

The FLOW trial [64] investigated the effects of semaglutide (1.0 mg/weekly) administered subcutaneously in patients with T2D and CKD. Eligible participants were adults diagnosed with T2D, with HbA1c ≤ 10% (86 mmol/mol), at high risk for CKD, and receiving a stable maximal labeled dose (or the maximal dose without unacceptable side effects) of renin-angiotensin (RAS) inhibitors [angiotensin-converting enzyme inhibitors (ACEis) or angiotensin-receptor blockers (ARBs)] [64]. Patients who were unable to receive RAS inhibitors because of side effects were eligible for inclusion [64].

CKD inclusion criteria were an eGFR of 50 to 75 ml/min/1.73 m^2^ and a urinary albumin-to-creatinine ratio (UACR) > 300 mg/g and < 5000 mg/g or an eGFR of 25 to 50 ml/min/1.73m^2^ and a UACR > 100 mg/g and < 5000 mg/g [64]. The use of SGLT2is and MRAs was permitted, and randomization was stratified according to SGLT2i use at baseline [64].

A total of 3533 participants, 68% of whom were at very high risk for kidney disease progression, kidney failure, CV events or death, were randomized 1:1 ratio to semaglutide (n = 1767) or placebo (n = 1766) [64]. The target dose of 1.0 mg of semaglutide was achieved gradually after 8 weeks of dose escalation, with the starting dose of semaglutide being 0.25 mg/week for 4 weeks, and 0.5 mg/week for another 4 weeks, followed by a maintenance dose of 1.0 mg/week throughout the remaining treatment period [64].

The primary outcome was the time to first occurrence of a composite of major kidney disease events: onset of a persistent ≥ 50% reduction in eGFR compared with baseline; onset of persistent eGFR < 15 mL/min/1.73 m^2^; initiation of chronic renal replacement therapy (dialysis or kidney transplantation), renal death or CV death (Table 3) [64]. Confirmatory secondary outcomes, assessed in a hierarchical order included an annual rate of change in eGFR (total eGFR slope), a composite of MACE outcomes consisting of nonfatal MI, nonfatal stroke or CV death, and death from any cause [64]. Additional supportive secondary endpoints were defined, a selection of which is shown in Table 3.

**Table 3:** Key information of the FLOW trial [64]

FLOW (64)		
Class & kidney and cardiovascular (CV) outcomes	Effect of semaglutide vs placebo (95% CI)*	p-value
*Primary outcome*		
Composite of major kidney disease events: onset of kidney failure^‡^, at least a 50% reduction in eGFR from baseline, or renal or CV death	0.76 (0.66 to 0.88)^§^	0.0003
*Confirmatory secondary outcomes*		
Mean annual rate of change in eGFR (ml/min/1.73m^2^)	− 2.19 vs. − 3.36 /1.16 (0.86 to 1.47)^#^	< 0.001
MACE (CV death, nonfatal MI, nonfatal stroke) (%)	0.82 (0.68 to 0.98)^§^	0.029
Deaths from any cause (%)	0.80 (0.67 to 0.95)^§^	0.01
*Supportive secondary outcomes of interest*		
Ratio of UACR at week 104 to UACR at baseline	0.68 (0.62 to 0.75)^§^	–
Mean change in body weight from baseline to week 104 (kg)	− 4.10 (− 4.56 to − 3.65)^#^	–
Mean change in HbA1c level from baseline to week 104 (% points)	− 0.81 (− 0.90 to − 0.72)^#^	–
Mean change in systolic BP from baseline to week 104 (mm Hg)	− 2.23 (− 3.33 to − 1.13)^#^	–
Mean change in diastolic BP from baseline to week 104 (mm Hg)	0.78 (0.16 to 1.41)^#^	–

Adverse events	Event rate (%) semaglutide vs. placebo group
*Serious adverse events*	49.6 vs. 53.8
CV disorders	15.4 vs. 18.1
Eye disorders	3.0 vs. 1.7
Diabetic retinopathy events	22.8 vs 22.5
*Adverse events leading to treatment discontinuation*	13.2 vs. 11.9
Gastrointestinal disorders	4.5 vs. 1.1

*Effect presented as HR or as between-group estimated difference.

§HR estimated with the use of Cox proportional hazard models.

#Between-group estimated difference.

‡Kidney failure defined by initiation of chronic renal replacement therapy (dialysis or kidney transplantation) or an eGFR < 15 ml/min/1.73m^2^.

*BP* Blood pressure, *CI* Confidence interval, *CV* Cardiovascular, *eGFR* Estimated glomerular filtration rate, *HbA1c* Glycated hemoglobin, *HR* Hazard ratio, *MACE* Major adverse cardiovascular event, *MI* Myocardial infarction, *UACR* Urinary albumin-to-creatinine ratio.

Time-to-first-event outcomes were analyzed with a stratified Cox proportional hazards model [64]. Continuous supportive secondary outcomes were assessed by analysis of covariance [64].

Major kidney disease events occurred less frequently in the semaglutide group (331 first events [5.8 per 100 patient-years]) than in the placebo group (410 first events [7.5 per 100 patient-years]), resulting in a 24% lower relative risk of the primary outcome in the semaglutide group (HR 0.76; 95% CI 0.66 to 0.88; p = 0.0003) (Table 3) [64]. Benefits in the semaglutide group were also observed for the three hierarchical secondary endpoints. The mean annual slope of the eGFR was significantly less steep in the semaglutide group than in the placebo group (− 2.19 vs. − 3.36 ml/ml/1.73 m^2^ per year (between-group estimated difference, 1.16; 95% CI 0.86 to 1.47; p < 0.001) (Table 3) [64], indicating a slower eGFR decrease. Fewer MACE events occurred in the semaglutide group (212 events [12.0%]) than in the placebo group (254 [14.4%]), an 18% difference (HR 0.82 (95% CI 0.68 to 0.98; p = 0.029) (Table 3) [64], and from the analysis of the data for death from any cause (227 events in the semaglutide group vs. 279 events in the placebo group (HR 0.80; 95% CI 0.67 to 0.9; p = 0.01) [64], it is estimated that patients receiving semaglutide have a 20% lower death risk.

Results for other supportive secondary outcomes are shown in Table 3. At 104 weeks, the UACR was reduced by 40% in the semaglutide group compared with 12% in the placebo group (HR 0.68; 95% CI 0.62 to 0.75) [64]; the mean reduction in body weight and HbA1c was 4.10 kg and 0.81%, respectively, greater in the semaglutide group compared to the placebo group [64]. However, concerning blood pressure (BP) differences were observed between the two groups. The mean reduction in systolic BP was 2.23 mmHg greater in patients receiving semaglutide (95% CI 1.13 to 3.33), but the mean reduction in diastolic BP was 0.78 mm Hg greater in patients receiving placebo (95% CI 0.16 to 1.41) (Table 3) [64].

Serious adverse events, including CV disorders, were reported in fewer participants receiving semaglutide than in the control group (877 [49.6%] vs. 950 [53.8%]) and included cardiac disorders (Table 3) [64]. Although the incidence of diabetic retinopathy events was similar in the two groups (Table 3), eye disorders were more common among participants receiving semaglutide than in those receiving placebo (53 [3.0%] vs. 30 [1.7%]) [64]. Adverse events leading to treatment discontinuation were more frequent in the semaglutide group and were mainly gastrointestinal in nature (Table 3) [64].

### Dual glucose-dependent insulinotropic polypeptide (GIP)/ glucagon-like peptide 1 (GLP-1) receptor agonists

#### SUMMIT [62]

The SUMMIT trial [62] evaluated CV outcomes in 731 patients with heart failure with preserved ejection fraction (HFpEF) and a BMI ≥ 30 who received subcutaneous tirzepatide once weekly up to a maximum tolerated dose (MTD) of 15 mg for at least 52 weeks. The median duration of follow-up was 104 weeks. Eligible patients were adults ≥ 40 years with chronic HF defined as New York Heart Association (NYHA) class II to IV, a LVEF ≥ 50%, and a BMI ≥ 30 kg/m^2^. Other important enrollment criteria included at least one of the following: an elevated N-terminal pro-B-type natriuretic peptide (NT-proBNP) level (> 200 pg/mL in patients with sinus rhythm or > 600 pg/mL in patients with atrial fibrillation), left atrial enlargement or elevated resting or exercise filling pressures, and HF decompensation within 12 months before baseline or an eGFR ≤ 70 ml/min/1.73 m^2^ [62]. Patients admitted to the study had a 6-min walk distance between 100 and 425 m and a KCCQ-CSS ≤ 80 [62].

After stratified randomization based on occurrence of HF decompensation before baseline, history of T2D, and BMI (< 35 or ≥ 35 kg/m^2^), patients were randomly assigned in a 1:1 ratio to receive tirzepatide or matching placebo in addition to standard therapy [62]. The starting dose of tirzepatide was 2.5 mg. If there were no unacceptable adverse events, the dose was increased by 2.5 mg every 4 weeks up to 15.0 mg per week after 20 weeks [62].

Two primary endpoints were specified: a composite of adjudicated CV death or worsening HF assessed in a time-to-first-event analysis, and the change from baseline to 52 weeks in the KCCQ-CSS [62]. If the effect on the primary outcome was significant for either primary endpoint, the following key secondary endpoints were to be analyzed: the change in the 6-min walk distance at 52 weeks, the percent change in body weight at 52 weeks, and the percent change in the hsCRP level at 52 weeks [62].

Primary endpoints were analyzed as the time to first event with the use of a Cox regression model. Between-group differences in changes in the KCCQ-CSS were analyzed with the use of the stratified Wilcoxon rank-sum test, and the Hodges–Lehmann method, with multiple imputation of missing data, was used to estimate the median difference regardless of patient adherence to the trial regimen, along with two-sided 95% confidence intervals [62].

At baseline, the mean age of the patients was 65.2 years, and the mean BMI was 38.3. The mean KCCQ-CSS was 53.5 points, the mean 6-min walk distance was 302.8 m, and 46.9% of patients had had a hospitalization or urgent care visit for worsening HF in the previous 12 months [62]. The median level of NT-proBNP was less than 200 pg/ml [62].

The composite endpoint (CV death or a worsening HF) occurred in 36 (9.9%) patients in the tirzepatide group and in 56 (15.3%) patients in the placebo group (HR 0.62; 95% CI 0.41 to 0.95; p = 0.026) (Table 4) [62]. Worsening HF events occurred in 29 (8.0%) patients in the tirzepatide group and in 52 (14.2%) patients in the placebo group (HR 0.54; 95% CI 0.34 to 0.85), and adjudicated CV death occurred in 8 (2.2%) patients and 5 (1.4%) patients, respectively (HR 1.58; 95% CI 0.52 to 4.83) [62]. However, it should be noted that two of the deaths in the tirzepatide group occurred after patients had stopped taking medication for more than 15 months [62]. At 52 weeks, the mean (± SD) change in the KCCQ-CSS was 19.5 ± 1.2 in the tirzepatide group as compared with 12.7 ± 1.3 in the placebo group (between-group difference, 6.9; 95% CI 3.3 to 10.6; p < 0.001) (Table 4) [62].

**Table 4:** Key information of the SUMMIT trial [62]

SUMMIT [62]		
Class & cardiovascular (CV) outcomes	HR or difference (95% CI)*	p-value
*Primary endpoints*		
Composite of adjudicated CV death or worsening HF events^◊^	0.62 (0.41 to 0.95)^§^	0.026
Change at 52 weeks in KCCQ-CSS	6.9 (3.3 to 10.6)^§^	< 0.001
*Key secondary endpoints*		
Change from baseline to week 52 in 6-min walk distance (m)	18.3 (9.9 to 26.7)^#^	< 0.001
Change at 52 weeks in body weight (%)	− 11.6 (− 12.9 to − 10.4)^#^	< 0.001
Change from baseline to week 52 in hsCRP level (%)	− 34.9 (− 45.6 to − 22.2)^#¥^	< 0.001
*Adjusted change at 52 weeks in physiological and laboratory measurements*		
NT-proBNP	0.90 (0.79 to 1.01)^∫^	–
Systolic BP (mm Hg)	− 4.7 (− 6.8 to − 2.5)^#^	–
Heart rate (beats/min)	2.8 (1.3 to 4.3)^#^	–

Adverse events	Event rate (%) tirzepatide vs. placebo group	
*Serious adverse events*	26.4 vs 25.6	
Heart failure	4.1 vs 8.2	
*Nonfatal adverse events leading to discontinuation of study medication*	6.3 vs. 1.4	
Gastrointestinal disorders	4.1 vs. 0	

*Values are HR estimated with the use of Cox proportional hazard models for the primary endpoints; all other values for key secondary endpoints and physiology and laboratory measurements are treatment between-group differences shown as medians, except for NT-proBNP.

^◊^Worsening HF events defined as worsening HF symptoms requiring hospitalization, intravenous HF drug therapy during urgent care or oral diuretic intensification.

§HR estimated with the use of Cox proportional hazard models.

#Between-group estimated difference.

¥Data data were log-transformed before the analysis.

∫NT-proBNP was measured in pg/ml and the ratio of the adjusted geometric mean ratios calculated. The data were log-transformed before the analysis.

*BP* Blood pressure, *CI* Confidence interval, *CV* Cardiovascular, *HF* Heart failure, *HR* Hazard ratio, *hsCRP* High-sensitivity C-reactive protein KCCQ-CSS, Kansas City Cardiomyopathy Questionnaire clinical summary score, *NT-proBNP* N-terminal pro-B type natriuretic peptide.

At 52 weeks, the mean percentage change in body weight was − 13.9% in the tirzepatide group and − 2.2% in the placebo group (between-group difference, − 11.6; 95% CI, − 12.9 to − 10.4; p < 0.001) (Table 4) [62]. There was a significant increase in the 6-min walking distance of 26.0 m in the tirzepatide group and 10.1 m in the placebo group (between-group difference, 18.3; 95% CI 9.9 to 26.7; p < 0.001) [62], and a significant decrease in the hsCRP percentage level of -38.8% and -5.9%, respectively (between-group change difference, -34.9; 95% CI -45.6 to -22.2; p < 0.001) (Table 4) [62]. The adjusted changes for other laboratory and physiological measurements of interest are shown in Table 4.

The number of serious adverse events was similar in the two groups (Table 4) [62]. However, heart failure occurred in 4.1% of the patients treated with tirzepatide, half of the cases of the placebo group (8.2%) [62]. Adverse events leading to discontinuation of the trial drug occurred in 23 (6.3%) patients in the tirzepatide group and in 5 (1.4%) patients in the placebo group (Table 4) and were mainly gastrointestinal in nature [62].

#### SURMOUNT-OSA trial [65]

The SURMOUNT-OSA trial [65] compared the efficacy and safety of the MTD of tirzepatide (10 mg or 15 mg) to placebo in adults living with moderate-to-severe OSA and obesity for 52 weeks. Two study populations were included in its placebo-master protocol. Participants who were unable or unwilling to use positive airway pressure (PAP) therapy were enrolled in Study 1, while participants who had been using PAP therapy for at least three consecutive months and planned to continue using it during the trial were enrolled in Study 2 [65].

PAP therapy reduces symptoms related to OSA and improves the apnea–hypopnea index (AHI), which is the number of apneas and hypopneas during an hour (hr) of sleep. AHI measurement by laboratory polysomnography was used to screen for eligible individuals for the SURMOUNT-OSA trial [65]. A total of 469 adults with an AHI of ≥ 15 events per hour and a BMI ≥ 30 (≥ 27 in Japan) was randomized 1:1 to receive MTD tirzepatide or placebo subcutaneously once weekly [65]. Tirzepatide was administered at a starting dose of 2.5 mg, which was increased by 2.5 mg every four weeks until the MTD was reached. Importantly, T1D or T2D were key exclusion criteria, as were a participant-reported change in body weight of more than 5 kg in the 3 months before screening, planned surgery for sleep apnea or obesity, a diagnosis of central or mixed sleep apnea, and major craniofacial abnormalities [65].

The primary endpoint was the change in AHI from baseline (Table 5) [65]. Key secondary endpoints were the percentage change in AHI; the percentage of participants with at least a 50% reduction in AHI; the percentage of participants with an AHI of less than 5 events per hour or with an AHI of 5 to 14 events per hour and an Epworth Sleepiness Scale (ESS) score of 10 or less; the percentage change in body weight; the change in hsCRP concentration; and the change in sleep apnea-specific hypoxic burden, a measure calculated from a polysomnographic study that includes the frequency, duration, and depth of oxygen saturation associated with the respiratory event (Table 5) [65].

**Table 5:** Key information of the SURMOUNT-OSA trial [65]

SURMOUNT-OSA [65]				
	Study 1		Study 2 (patients using PAP therapy)	
Class & metabolic and cardiovascular (CV) outcomes	Estimated treatment difference or relative risk (95% CI)*	p-value	Estimated treatment difference or relative risk (95% CI)*	p-value
*Primary endpoint*				
Change in AHI (95% CI)—no. of events/hr	− 20.0 (− 25.8 to − 14.2)	< 0.001	− 23.8 (− 29.6 to − 17.9)	< 0.001
*Key secondary endpoints*				
Percent change in AHI (95% CI)	− 47.7 (− 65.8 to -29.6)	< 0.001	− 56.2 (− 73.7 to − 38.7)	< 0.001
Reduction of ≥ 50% in AHI events at week 52-no. (%)	3.3 (2.1 to 5.1)$\phi$	< 0.001	3.1 (2.1 to 4.5)	< 0.001
AHI of < 5 or AHI of 5 to 14 with ESS ≤ 10 at week 52-no. (%)	2.9 (1.8 to 4.8)$\phi$	< 0.001	3.3 (2.0 to 5.4)	< 0.001
Percent change in body weight (95% CI)	− 16.1 (− 18.0 to -14.2)	< 0.001	− 17.3 (− 19.3 to − 15.3)	< 0.001
Change in hsCRP concentration at week 52 (95% CI) (mg/l)	− 0.7 (− 1.2 to − 0.2)	0.004	− 1.0 (− 1.6 to − 0.5)	< 0.001
Change in sleep apnea–specific hypoxic burden at week 52 (95% CI) (% min/hr)	− 70.1 (− 90.9 to -49.3)	< 0.001	− 61.3 (− 84.7 to − 37.9)	< 0.001
Change in systolic BP at week 48 (95% CI) (mm Hg)	− 7.6 (− 10.5 to − 4.8)	< 0.001	− 3.7 (− 6.8 to − 0.7)	0.02

Most reported adverse events	Event rate (%) tirzepatide vs. placebo group			
Diarrhea	26.3 vs. 12.5		21.8 vs. 8.8	
Nausea	25.4 vs. 10.0		21.8 vs. 5.3	
Vomiting	17.5 vs. 4.2		9.2 vs. 0.9	
Constipation	15.8 vs. 2.5		15.1 vs. 4.4	

*Differences between groups are presented as estimated treatment differences unless otherwise stated. Estimated treatment differences for the secondary endpoints are the differences in the least-squares mean changes.

$\phi$Relative risk. Relative risks were calculated using g-computation methods.

*AHI* Apnea–hypopnea index, *BP* Blood pressure, *CI* Confidence interval, *ESS* Epworth Sleepiness Scale, *hsCRP* High-sensitivity C-reactive protein, *PAP* Positive airway pressure.

Both primary and secondary endpoints results were assessed and reported with the use of the treatment-regimen estimand [65]. For Study 1 treatment-regimen estimand, the change in AHI at week 52 was − 25.3 events/hr with tirzepatide and -5.3 events/hr with placebo, for an estimated treatment difference of − 20.0 events/hr (95% CI − 25.8 to − 14.2; p < 0.001) (Table 5). For Study 2 treatment-regimen estimand, the change in AHI at week 52 was -29.3 events/hr with tirzepatide and − 5.5 events/hr with placebo, for an estimated treatment difference of − 23.8 events/hr (95% CI − 29.6 to − 17.9; p < 0.001) (Table 5) [65].

As for the key secondary endpoints, participants treated with tirzepatide in both studies experienced significant reductions in AHI compared to placebo: − 47.7 (95% CI − 65.8 to − 29.6; p < 0.001) in Study 1 and − 56.2 (95% CI − 73.7 to − 38.7; p < 0.001) in Study 2 (Table 5) [65]. In addition, 61.2% and 72.4% of participants receiving tirzepatide in Study 1 and Study 2, respectively, achieved a 50% or greater reduction in AHI events at week 52 [65]. Notably, 42.2% of the tirzepatide group in Study 1 and 50.2% in Study 2 achieved an AHI of less than 5 events or an AHI of 5 to 14 events with an ESS score of 10 or less at week 52 [65]. The calculated relative risks for these secondary endpoints are shown in Table 5. Improvements in body weight, systolic blood pressure, hypoxic burden and hsCRP were also observed in the tirzepatide-treated arms of both studies compared to placebo (Table 5) [65].

The SURMOUNT-OSA clinical trial also included patient-reported outcomes assessments (PROs), by measuring the scores of the Patient-Reported Outcomes Measurement Information System (PROMIS) Short Form Sleep-related Impairment 8a (PROMIS-SRI) and PROMIS Short Form Sleep Disturbance 8b (PROMIS-SD) scales [65]. Based on these scores, positive effects of tirzepatide on participants’ sleep-related functioning and sleep disturbance were observed.

Adverse events were reported in 79.8% of the tirzepatide group and 76.7% of the placebo group in Study 1, and in 83.2% of the tirzepatide group and in 72.8% of the placebo group in Study 2 [65]. The most common events in those on tirzepatide compared with placebo, respectively, were diarrhea (26.3% vs 12.5%), nausea (25.4% vs 10.0%) and vomiting (17.5% vs 4.2%) in Study 1, and diarrhea (21.8% vs 8.8%), nausea (21.8% vs 5.3%) and constipation (15.1% vs 4.4%) in Study 2 (Table 5) [65]. In general, the severity of these gastrointestinal events was mild to moderate [65]. A total of 9 patients on tirzepatide (5 in Study1 and 4 in Study2) and 10 patients on placebo (2 in Study1 and 8 in Study2) discontinued treatment due to adverse events. A total of 35 serious adverse events (7.5%) were reported, with no deaths [65].

### Nonsteroidal mineralocorticoid receptor antagonists (nsMRAs)

#### FINEARTS-HF [63]

The FINEARTS-HF trial [63] evaluated the efficacy and safety of finerenone in addition to usual therapy in 6001 patients with HF with mildly reduced or preserved ejection fraction (LVEF ≥ 40%) over a median follow-up of 32 months. Patients 40 years of age or older with symptomatic HF, LVEF ≥ 40%, evidence of structural heart disease, and elevated natriuretic peptide levels were eligible [63]. Patients were randomly assigned 1:1 to finerenone (n = 3003) and placebo (n = 2998). Depending on the baseline eGFR, finerenone was administered at a maximum dose of 20 mg or 40 mg daily [63].

The primary outcome was a composite of total worsening HF events and CV death (Table 6) [63]. A worsening HF event was defined as a first or recurrent unplanned hospitalization or urgent visit for HF. Secondary outcomes were defined as total worsening HF events; the change from baseline in the overall symptom score on the Kansas City Cardiomyopathy Questionnaire (KCCQ-OSS) at months 6, 9, and 12; improvement in the NYHA functional class at month 12; and a kidney composite outcome (a composite of a sustained decrease in the eGFR of ≥ 50%, a sustained decline in the eGFR to < 15 ml/min/1.73m^2^, or the initiation of long-term dialysis or kidney transplantation) and evaluated hierarchically in a time-to-event analysis [63]. Death from any cause was also assessed.

**Table 6:** Key information of the FINEARTS-HF trial [63]

FINEARTS-HF [63]		
Class & cardiovascular (CV) outcomes	Effect of finerenone vs placebo (95% CI)*	p-value
*Primary outcome*		
Composite of total worsening HF events^◊^ and CV death	0.84 (0.74 to 0.95)^#^	0.007
*Secondary outcomes*		
Mean change from baseline in KCCQ-OSS at 6, 9, and 12 months (points)	1.6 (0.8 to 2.3)^¤^	< 0.001
Improvement in NYHA functional class at 12 months	1.01 (0.88 to 1.15)^¥^	–
Kidney composite outcomes^†^	1.33 (0.94–1.89)^§^	–
Death from any cause	0.93 (0.83 to 1.06)^§^	–

Adverse events	Event rate (%) finerenone vs. placebo group
*Serious adverse events*	38.7 vs 40.5
Serum creatinine level ≥ 3.0 mg/dl	2.0 vs. 1.2
Serum potassium level> 5.5 mmol/l	14.3 vs. 6.9
Serum potassium level > 6.0 mmol/l	3.0 vs. 1.4
Serum potassium level < 3.5 mmol/l	4.4 vs. 9.7
Investigator-reported hyperkalemia	9.7 vs 4.2
Hyperkalemia that led to hospitalization	0.5 vs. 0.2
Hyperkalemia that led to death	0 vs. 0

*Effect presented as HR or as between-group estimated difference.

#Rate ratio.

§HR estimated with the use of Cox proportional hazard models.

¤Between-group difference.

¥Odds ratio.

^◊^Worsening HF events were defined as a first or recurrent unplanned hospitalization or urgent visit for HF.

†The kidney composite outcome was defined as a composite of a sustained decrease in eGFR) ≥ 50%, sustained decline eGFR to less than 15 ml/min/1.73 m^2^ of body surface area, or the initiation of long-term dialysis or kidney transplantation, assessed in a time-to-event analysis.

*BP* Blood pressure, *CI* Confidence interval, *CV* Cardiovascular, *HF* Heart failure, *HR* Hazard ratio, *KCCQ-OSS* Kansas City Cardiomyopathy Questionnaire Overall Summary Score, *NYHA* New York Heart Association.

The primary analysis was performed according to an intention-to-treat approach with the semiparametric proportional rates method [77], stratified according to geographic region and baseline LVEF (< 60% or ≥ 60%) [63]. Primary outcome events occurred in 624 (20.7%) patients in the finerenone group, and in 719 (24.0%) patients in the placebo group (rate ratio, 0.84; 95% CI 0.74 to 0.95; p = 0.007) (Table 6) [63]. The total number of worsening HF events was 842 (28%) in the finerenone group and 1024 (34.1%) in the placebo group (rate ratio, 0.82; 95% CI 0.71 to 0.94; p = 0.006) [63]. CV death occurred in 242 (8.1%) patients in the finerenone group and 260 (8.7%) patients in the placebo group (HR 0.93; 95% CI 0.78 to 1.11) [63].

For the secondary outcomes, the least-squares mean change ( ±) from baseline in the KCCQ-OSS, which was estimated as a common treatment effect across months 6, 9, and 12, was 8.0 ± 0.3 points in the finerenone group and 6.4 ± 0.3 points in the placebo group (between-group difference, 1.6; 95% CI 0.8 to 2.3; p < 0.001) and improvement in NYHA functional class was observed in 557 (18.6%) patients receiving finerenone and 553 patients (18.4%) in the placebo group (odds ratio, 1.01; 95% CI 0.88 to 1.15). A composite of kidney outcomes was reported in 75 (2.5%) of the patients in the finerenone group and 55 (1.8%) patients in the placebo group, corresponding to an HR of 1.33 (95% CI 0.94 to 1.89) (Table 6) [63]. Death from any cause occurred in 491 (16.4%) patients in the finerenone group and 522 (17.4%) patients in the placebo group (HR 0.93; 95% CI 0.83 to 1.06) [63]. In a prespecified sensitivity analysis, a composite of first worsening HF event or CV death was evaluated in a time-to-event analysis and was observed in 624 (20.8%) and 719 (24.0%) of patients in the finerenone and placebo groups, respectively (HR 0.84; 95% CI 0.76 to 0.94) [63].

Serious adverse events occurred in 1157 (38.7%) patients in the finerenone group and 1213 (40.5%) patients in the placebo group (Table 6) [63]. Increases in creatinine levels were more common with finerenone than with placebo (Table 6) [63]. Finerenone was also associated with both an increased risk of hyperkalemia (Table 6) [63], with potassium levels greater than 6.0 mmol/l occurring in 86 (3.0%) patients in the finerenone group and 41 (1.4%) patients in the placebo group, and a reduced risk of hypokalemia (Table 6) [63]. However, no episodes of hyperkalemia led to death [63].

## Key topics discussed during the 10th CVOT summit

### Examples of guidelines

#### Guidelines development: moving toward precision medicine

Clinical practice guidelines (CPGs) are formulated based on collected medical evidence gathered from case series/reports, case–control studies, cohort studies, randomized controlled trials (RCTs), systematic reviews, and meta-analyses [78]. The different sources of evidence are ranked according to their strength in a hierarchical pyramid system [78], with RCTs, systematic reviews, meta-analyses, and in the last 20 years network (or multiple treatment) meta-analyses (NMAs) at the top. NMAs can be narrowed down to assess specific populations, which might be useful for defining which therapies show increased efficacy and reduced side effects in the context treatment of heterogeneous conditions such as diabetes [79] or obesity. However, to align with the principles of precision medicine and person-centered care, NMAs should address several clinical gaps, including refining methodologies to account for heterogeneity and effect modifiers, embracing real-world evidence (RWE), and incorporating patient-level data. In this regard, the Taskforce of the Guidelines Workshop is committed to drawing attention to the disparities, treatment inequities, and impact of social determinants of health (SDOH) in diabetes, obesity, CVD, and CKD outcomes [80], as well as to considering the value of collecting PROs in these patients in a standardized manner also for clinical guideline development. Recently, a consensus paper on the standardization of PROs in diabetes research has been published [81]. However, it is also important to collect PROs in clinical practice to measure not only treatment satisfaction, but also its impact on the health-related quality of life of people living with diabetes, obesity, CVD, and CKD. Only then can personalized medicine be delivered and the promise of precision medicine fulfilled.

### Update on chronic kidney disease (CKD) and metabolic dysfunction-associated steatotic liver disease (MASLD) management guidelines

In 2024, the CKD work group of Kidney Disease: Improving Outcomes (KDIGO) updated its clinical practice guideline for the evaluation and management of this disease [82]. Besides a general recommendation for the use of SGLT2is as first-line therapy in addition to lifestyle modifications in most patients, the guideline recommends the use of externally validated risk prediction equations to calculate the absolute risk of kidney failure in individuals with CKD and an eGFR category of G3 to G5 [82]. Assessment of eGFR and UACR in people at risk for CKD is also emphasized in the guideline to identify persons with CKD, to avoid potentially nephrotoxic medications; to adjust the dosage of drugs with a narrow therapeutic range; to reduce the use of volume or contrast agents; to minimize the risk of acute chronic kidney injury; and to help decide when to initiate kidney replacement therapy [82].

Also, this year, the European Association for the Study of the Liver (EASL), the European Association for the Study of Diabetes (EASD), and the European Association for the Study of Obesity (EASO) published a joint guideline providing an update on definitions, prevention, screening, diagnosis and treatment for MASLD [83]. MASLD, previously termed nonalcoholic fatty liver disease (NAFLD), is defined as steatotic liver disease (SLD) in the presence of one or more cardiometabolic risk factor(s) and the absence of harmful alcohol intake [83]. The spectrum of MASLD is broad and includes steatosis, metabolic dysfunction-associated steatohepatitis (MASH, previously NASH), fibrosis, cirrhosis, and MASH-related hepatocellular carcinoma (HCC). Guidelines recommend screening for MASLD with liver fibrosis either in individuals with T2D; abdominal obesity and ≥ 1 additional metabolic risk factor(s); or abnormal liver function tests [83]. A strategy for non-invasive assessment of the risk for advanced fibrosis and liver-related outcomes is proposed [83]. The amount of alcohol intake, the drinking pattern, and the type of alcohol consumed should be assessed in all individuals with SLD using a detailed medical history, psychometric instruments, and/or validated biomarkers [83]. In addition to dietary and lifestyle modifications, pharmacologic treatment of comorbidities is recommended. Preferred options for the treatment of comorbidities are GLP-1 RAs, tirzepatide (dual GIP/GLP-1 RA), SGLT2is, metformin or insulin (in case of decompensated cirrhosis) for T2D; statins for dyslipidemia; and GLP-1 RAs, tirzepatide (dual GIP/GLP-1 RA) for obesity [83]. For fibrosis stages F2/F3, treatment with resmetirom, a liver-directed thyroid hormone receptor beta agonist, is recommended [83]. Resmetirom has been approved as a MASH-targeted therapy in the US, while its approval in Europe is pending. Bariatric surgery is also an option for individuals with MASLD and obesity [83].

### Advances in the management of heart failure (HF) and chronic kidney disease (CKD)

Reducing morbidity (i.e., reducing symptoms, improving health-related quality of life and functional status, and reducing hospitalization rate) and mortality are the goals of HF management. ACEIs, ARBs, neprilysin inhibitors, beta-blockers, MRAs, and SGLT2i are the basis of pharmacotherapy for HF with reduced ejection fraction (HFrEF) [84]. SGLT2i are also recommended for the treatment of mildly reduced ejection fraction (HFmrEF) and HFpEF [84].

In 2023 and 2024, the results of the trials with semaglutide (STEP-HFPEF [42] and STEP-HFPEF-DM [61]), tirzepatide (SUMMIT [62]), and finerenone (FINEARTS-HF [63]) were published, and these classes of drugs are also being considered as future pillars of HFpEF therapy. A proposed algorithm for the treatment of HFpEF, which includes SGLT2i, nsMRA and, in the presence of obesity, semaglutide/tirzepatide as basic therapeutics to reduce clinical events and to improve symptoms, and physical limitations, was presented at the European Society of Cardiology (ESC) Congress 2024 and the CVOT Summit 2024 (Mikhail Kosiborod, oral presentation).

CKD is a global concern that presents significant challenges for disease management. The total prevalence of CKD is projected to rise to 436.6 million cases by 2027 (an increase of 5.8% from 2022), with most cases (∼80%) remaining undiagnosed [85]. SGLT2i are now recommended as first-line therapy, and finerenone is recommended in addition to SGLT2i in patients with CKD and T2D [82]. However, the novel results of the FLOW trial [64], stimulated the debate on the role of GLP-1 RAs for CKD management in guidelines. A detailed analysis of the FLOW trial [64] shows that the early dip in eGFR slope induced by SGLT2i, usually only temporary, may be perceived as concerning at the start of treatment, and appears to be attenuated by semaglutide [86].

Another promising class of medications in CKD are aldosterone synthase inhibitors (ASIs). The RAS system and aldosterone are important for the regulation of blood pressure and CV function. Elevated levels of aldosterone can lead to organ damage with detrimental effects on the heart and kidney and are associated with worse outcomes in patients with HF and CKD [87].

Recently, a phase II trial investigated the efficacy of vicadrostat, an ASI, in addition to empagliflozin [10] or placebo in patients with CKD [88]. Vicadrostat at doses of 3 mg, 10 mg or 20 mg or placebo was given once daily for 14 weeks to adults with CKD (n = 714), with or without T2D, on stable background treatment with an ACEi or ARB for more than 4 weeks prior to screening, with an eGFR of 30 to 90 ml/min/1.73 m^2^, and a UACR of 200 to 5000 g/g [88]. Regardless of the addition of empagliflozin, all doses of vicadrostat reduced aldosterone exposure [88]. Furthermore, vicadrostat was shown to reduce UACR by up to 40% in patients with CKD after 8 weeks of empagliflozin run in. Since UACR reduction is associated with improved outcomes in patients with HFpEF [89], it might be that the combination of vicadrostat and empagliflozin may be beneficial for this population. Vicadrostat is being investigated in the phase III EASi-KIDNEY trial (ClinicalTrials.gov Identifier: NCT06531824) for the treatment of CKD and HF (LVEF ≥ 40%).

Baxdrostat, another ASI, has been shown to significantly reduce systolic BP in patients with treatment-resistant hypertension (BP ≥ 130/80 mmHg) who were on stable doses of at least three antihypertensive agents, including a diuretic [90]. The efficacy and safety of baxdrostat in combination with dapagliflozin on CKD progression in patients with CKD and hypertension is being evaluated in a phase III trial (NCT06268873).

The treatment of CKD in T1D is particularly challenging. FINE-ONE (ClinicalTrials.gov Identifier: NCT05901831) with finerenone and SUGARNSALT (ClinicalTrials.gov Identifier: NCT06217302) with sotagliflozin are the first clinical trials in 30 years to seek an indication for a new treatment for T1D and CKD. Additionally, the ATTEMPT trial (ClinicalTrials.gov Identifier: NCT04333823) is evaluating the effectiveness of dapagliflozin to optimise diabetes control and prevent early subclinical kidney complications in adolescents with T1D. The results will be particularly interesting with regard to the ongoing debate on whether SGLT2i should be used in patients with T1D considering the risk for diabetic ketoacidosis.

Other interventions exploring the potential of endothelin receptor antagonists and soluble guanylyl cyclase stimulators and activators, as well as incretins, may follow.

### Organ crosstalk and cardio-kidney-metabolic (CKM) syndrome

Accumulating evidence on the epidemiologic link between diabetes, obesity, and CVD, the understanding of the interplay among metabolic risk factors, CKD, and the CV system, and their impact on morbidity and mortality, led the American Heart Association (AHA) to define the cardiovascular-kidney-metabolic (CKM) syndrome. CKM is defined as a health disorder attributable to the connections among obesity, diabetes, CKD, and CVD, including HF, atrial fibrillation, coronary heart disease, stroke, and peripheral artery disease [91]. The CKM syndrome includes those at risk for CVD and those with existing CVD [91].

Nearly every major organ system is affected by CKM syndrome, with associated clinical challenges including kidney failure, premature cognitive decline, MASLD, OSA, and an increased risk of cancer. However, the greatest clinical impact of CKM syndrome regarding morbidity and premature mortality is through the disproportionate burden of CVD [91].

CKM is a progressive disorder that is subdivided into stages. Stage 1, excess or dysfunctional adipose tissue; Stage 2, metabolic risk factors and CKD; Stage 3, subclinical CVD in CKM syndrome; and Stage 4, clinical CVD in CKM syndrome [91].

### Micro-and nanoplastics as an emerging risk factor for cardiovascular disease (CVD)

Despite good control of risk factors, CV mortality remains very high, representing 32% of all global deaths [92]. An increased incidence of coronary artery disease has been linked to air pollution, which was estimated to cause 9 million deaths worldwide in 2019, of which 62% were due to CVD and 31.7% to coronary artery disease [93].

In particular, micro- and nanoplastics (MNPs), which are a major source of environmental pollution, seem to trigger CVD [94], as evidence is emerging describing the accumulation of small plastic particles in various organs and tissues of the body, mainly through ingestion and inhalation [95].

In a recent study, MNPs were found in the carotid artery plaque of 58% (n = 150) of patients with asymptomatic carotid artery disease [96]. These patients were found to be at higher risk for myocardial infarction (MI), stroke, or death from any cause than those in whom these substances were not detected (HR 4.53; 95% CI 2.00 to 10.27; p < 0.001) [96].

### Role of primary care in diabetes education and cardio-kidney-metabolic (CKM) evaluation

Despite advances in diabetes treatment modalities, glycemic and cardiometabolic outcomes continue to decline worldwide. Diabetes self-management education and support (DSMES) have been shown to be effective in improving outcomes and are a vital component of treatment. The benefits of structured DMES include reductions in HbA1c levels and the frequency of hypoglycemia and hyperglycemia episodes. Additionally, it enhances treatment satisfaction and adherence, blood glucose self-monitoring, emotional well-being, quality of life, and healthy behaviors (exercise, diet, smoking cessation). It also empowers individuals living with diabetes by giving them the confidence to manage their condition successfully and improve their overall health [97]. The effectiveness of DSMES should be evaluated as part of routine care at diagnosis, annually and/or when treatment targets are not being met, when complicating factors (medical, physical, psychosocial) develop, and when life and care transitions occur [98]. Primary care can play a pivotal role in supporting patients to enroll in DSMES.

Primary care also has a central role in CKM, assessing chronic inflammatory conditions, high burden of adverse SDOH, ethnicity, mental health disorders, sleep disorders, sex-specific risk-enhancing factors (premature menopause, polycystic ovarian syndrome, erectile dysfunction), and family history of kidney failure and diabetes, which are essential to determine the stage of progression of this disease [91].

### Early detection of type 1 diabetes (T1D) and strategies to delay its onset

Approximately 5–10% of all people with diabetes have T1D [99], an autoimmune disease in which the insulin-producing beta cells in the pancreas are destroyed. T1D can be triggered by environmental factors such as viral infections, diet, and growth (in children) in genetically susceptible individuals [100]. Typically, individuals diagnosed with T1D will be lifelong dependent on exogenous insulin. However, individuals in early stage 3 (glucose levels consistent with the definition of diabetes mellitus) do not immediately require insulin [101]. This means that there is a window of opportunity to intervene and delay insulin therapy as long as T1D is detected in the presymptomatic (stages 1 and 2) or early clinical stages (stage 3).

In stage 1, individuals are normoglycemic and positive for autoantibodies to ß-cell antigens. The disease then progresses to asymptomatic stage 2 with dysglycemia. When there is a family history of T1D, autoantibody screening can identify children in the early stages of T1D. Unfortunately, these cases represent only 10% of people with T1D [102]. There are other risk factors, like genetic factors and a family history of other autoimmune diseases, which should be considered.

Screening for early-stage T1D has the benefit of reducing the likelihood of stage 3 T1D ketoacidosis, which is associated with morbidity, mortality, and long-term consequences. Furthermore, it gives the patient and the family time to educate and prepare for disease progression, and enables living longer with insulin-free T1D avoiding the risk of hypoglycemia. These considerations have recently been published in a consensus document [102]. Certainly, screening for T1D also entails a psychological burden, which is why adequate accompanying psychological care should be ensured.

Delaying the onset of clinical T1D is possible with immunotherapy. Teplizumab has been approved for delaying the onset of stage 3 T1D in individuals at stage 2 [103], but research is also focusing on delaying the onset of diabetes already at stage 1. Abatacept treatment for 1 year had some effect on preserving insulin production in at-risk relatives in stage 1, but did not meet the primary endpoint of delaying progression to stage 2/3 [104].

### Diabetes, obesity, and the heart

A subset of patients with diabetes and HF may have a myocardial disorder due to the diabetes (formerly diabetic cardiomyopathy), causing systolic and/or diastolic dysfunction [105]. Presently it is accepted that myocardial dysfunction occurs mostly with diabetes in association with hypertension, obesity, coronary artery disease, and CKD [105].

The underlying pathogenesis is partially understood. Systemic factors, including hyperglycemia, insulin resistance, hyperlipidemia, excessive production of advanced glycation end-products (AGEs), activation of renin–angiotensin–aldosterone system (RAAS) and autonomic dysregulation promote pathological cellular and molecular processes in the myocardium leading to uncoupling of mitochondrial proteins and mitochondrial dysfunction with consequent increase in oxidative stress [106]. Circulating metabolites indicating dysregulated mitochondrial fatty acid oxidation were found to be elevated in individuals with T2D who subsequently experience MACE, and have recently been proposed as biomarkers for CV risk prediction [107].

### Cardiovascular autonomic neuropathy (CAN)

Cardiovascular autonomic neuropathy (CAN) is a major microvascular complication of T1D and T2D, defined as the impairment of CV autonomic control in patients with established diabetes, after exclusion of other causes [108]. It is characterized by impaired feeling of chest pain normally related to myocardial ischemia, orthostatic hypotension, resting tachycardia, impaired exercise tolerance, and abnormal blood pressure regulation, but it may also remain asymptomatic [109].

Systemic factors are involved in the pathogenesis of CAN, including hyperglycemia, oxidative stress, inflammation, microvascular damage, dyslipidemia, and insulin resistance [110]. Glucose control is of paramount importance in preventing its development, since high blood glucose levels lead to the formation of AGEs, which damage nerves and blood vessels [110].

### Advances in continuous glucose monitoring (CGM) and automated insulin delivery (AID)

CGM is now considered the standard of care for people with T1D or for people with T2D receiving intensive insulin therapy, but evidence is accumulating supporting the benefits of CGM in T2D patients treated with basal or multiple-day insulin injections or initiating GLP-1 RA therapy [111]. RCTs and observational studies do indeed confirm the clinical value of CGM in T2D regardless of treatment [112]. CGM has also proven to be valuable in patients with T2D and established CVD [113]. Severe hypoglycemia is strongly associated with an increased risk of macro- and microvascular events, and death in people with T2D [114, 115]. In people with T2D and recent MI, CGM significantly decreased hypoglycemic exposure compared with self-monitoring of blood glucose [113]. Hence, extending the use of CGM to T2D could minimize hypoglycemia, allow efficient adjustment and escalation of therapies [112], and may possibly improve CV outcomes, which has to be investigated [112].

In recent years, automated insulin delivery (AID) systems have become available, expanding the ability to achieve the recommended time in range (TIR) and blood glucose levels that are particularly challenging for people with T1D during physical activity and exercise. A position paper from EASD and the International Society for Pediatric and Adolescent Diabetes (IASP) has just been published that provides guidance on the use of all currently available AID systems around physical activity and exercise [116, 117].

### Incretin therapies in obesity, liver disease and obstructive sleep apnea (OSA)

Obesity (ectopic fat) increases the risk of CVD and premature death. Several years ago, it was proposed to consider epicardial fat as part of the ectopic fat concept [118]. Epicardial adipose tissue can affect heart function via inflammation, neural dysregulation, and fibrosis [119]. Lipogenesis also triggers fibrosis via inflammation in the liver in advanced forms of MASLD. Trials with incretin coagonists have shown dramatic reductions in body weight and fat, and improvement in cardiovascular outcomes, and now semaglutide is being evaluated for improvement of liver fibrosis in MASH and moderate to advanced liver fibrosis (stage or 2 or 3) in the phase II ESSENCE trial [120].

New data have also been published from phase 2 trials with tirzepatide (dual GIP/GLP-1 RA) [121] and survodutide (dual Glucagon/GLP-1 RA) [122] with improvement of fibrosis in F2/F3 stages. Whether incretin mimetics have direct, independent effects on MASH, or whether they affect pathophysiology through improvements in weight, insulin resistance, and glycemic control remains unclear.

Important advances have also been made with incretin agonists for the treatment of OSA as demonstrated by the SURMOUNT-OSA trial [65]. OSA is characterized by repetitive pharyngeal collapse during sleep resulting in apneas and hypopneas, with consequent hypoxemia, hypercapnia, and recurrent arousals, and is accompanied by clinically relevant symptoms, such as excessive daytime sleepiness, and is an independent risk factor for CVD [123].

Treatment with continuous PAP is recommended, but treatment adherence is suboptimal: 46–83% of individuals fail to adhere [124]. Approximately 50% of the patients complained of at least one side effect due to the nasal mask (facial allergy, air leaks, nasal ridge abrasions) [124]. Thus, the recent findings of SURMOUNT-OSA [65] offer new therapeutic options.

### Diet and nutrition under incretin-based therapy

Dietary modification is essential in the management of T2D and obesity. Globally, dietary recommendations are largely consistent with healthy components like nutritional fiber, vegetable, legume and fruit intake, while also advocating for adequate high‐quality protein and low-fat consumption. However, there is an interindividual variability in metabolic response to specific diets [125], based on genetics, dietary habits, eating patterns, physical activity, the microbiota, and the metabolome [125]. Precision nutrition based on metabolic phenotypes may increase the effectiveness of interventions. Recently, modulation of macronutrient composition within the dietary guidelines based on tissue-specific insulin resistance phenotypes has been demonstrated to improve cardiometabolic health [126].

As precision nutrition makes its way into nutrition research, other nutritional considerations are being made in the treatment of diabetes and obesity with incretin-based therapies. Particularly, the shift in food preferences and a reduced protein intake are discussed in this regard [127]. Incretin mimetics include liraglutide and semaglutide (GLP-1RAs), tirzepatide (GLP-1 and GIP receptor dual agonist), and retatrutide (GLP-1, GIP, and glucagon receptor triple agonist) which induce approximately 15–24% weight loss in adults with overweight and obesity [128], alongside beneficial impacts on cardiometabolic factors including glycemia, MASLD/MASH and OSA. However, the most common cause of treatment discontinuation in clinical trials with these drugs are gastrointestinal disorders, with nausea, vomiting, diarrhea, or constipation among the most frequent adverse effects reported. Furthermore, these peptides with incretin agonist activity may cause rapid and significant loss of the lean body mass (∼10% or ∼6 kg), comparable to a decade or more of aging [128]. Preservation of muscle mass and function during aging is crucial to avoid sarcopenia and frailty, which are strongly associated with morbidity and mortality, and retaining of lean mass during incretin therapy could blunt the regain of body weight (and fat) upon discontinuation of obesity pharmacotherapy. However, chronic treatment is recommended.

There is limited prospective data on the impact of physical activity or resistance training on body composition outcomes in people treated with incretin-based therapies. Exercise combined with liraglutide after an 8-week low-calory diet was found to increase lean mass by 0.8% compared with liraglutide (0.0%) and exercise alone (3.4%), and to decrease body fat mass by 3.5%, almost the double of liraglutide (1.6%) and exercise alone (1.8%) [129].

Besides physical activity, attention also should be paid to social and emotional health [130]. Changing dietary habits is not easy. Therefore, assessment of baseline dietary patterns and preferences is important for individualized treatment, as well as ongoing monitoring to facilitate early detection and management of gastrointestinal symptoms or inadequate nutrient or fluid intake. The focus on dietary management with incretin-based therapies should be on nutritional balance rather than restriction [130]. Some evidence-based recommendations have recently been published [130]. During weight loss, energy intake of 1200–1500 kcal/day for women, and 1500–1800 kcal/day for men are considered as safe [130]. Protein intake should be up to > 1.5 g/kg body weight per day, and carbohydrate and fat intake should be 135–290 g/day and 25–70 g/ day, respectively [130]. Fiber intake should be 21–25 g/day for women and 30/38 g/day for men [130]. Drinking at least 2 to 3 L per day is recommended, preferably water, low-calorie beverages, or nutrient-dense beverages [130]. These recommendations should be tailored to individual health profiles, particularly in the presence of underlying medical conditions and risks for malnutrition. Clinicians prescribing incretin-based therapies are encouraged to conduct a thorough initial health assessment and consider referring at-risk patients to a registered dietitian for comprehensive evaluation and ongoing follow-up.

## Conclusions

The 10th CVOT Summit: Congress on Cardiovascular, Kidney, and Metabolic Outcomes provided an interactive and multidisciplinary platform to discuss key results from recently published trials with SGLT2i (EMPACT-MI), GLP-1 RA (STEP-HFpEF-DM and FLOW), dual GIP/GLP-1 RA (SUMMIT and SURMOUNT-OSA) and nsMRA (FINEARTS-HF). These and other aspects including T1D and CGM were discussed. New data on obesity, including the nutritional aspects during incretin-based therapies, as well as diagnosis and management of liver disease were also discussed by a broad audience of specialists and primary care physicians. The 11th CVOT Summit will be held virtually on November 20–21, 2025 (http://www.cvot.org).

## Data Availability

No datasets were generated or analyzed during the current study.

## Abbreviations

ACEis: Angiotensin-converting-enzyme inhibitors
ADA: American diabetes association
AGE: Advanced glycation end product
AGP: Ambulatory glucose profile
AHI: Apnea–hypopnea index
ALA: Alpha-lipoic acid
ARBs: Angiotensin-receptor blockers
ASCVD: Atherosclerotic cardiovascular disease
ASI: Aldosterone synthase inhibitor
BMI: Body mass index
BP: Blood pressure
CAN: Cardiovascular autonomic neuropathy
CGM: Continuous glucose monitoring
CI: Confidence interval
CKD: Chronic kidney disease
CKM: Cardio-kidney-metabolic
CRP: C-reactive protein
CV: Cardiovascular
CVD: Cardiovascular disease
CVOT: Cardiovascular outcome trial
DALYs: Disability-adjusted life years
DKD: Kidney disease due to diabetes
DPN: Diabetic peripheral neuropathy
DPP-4i: Di-peptidyl peptidase 4 inhibitor
DSPN: Diabetic sensorimotor polyneuropathy
eGFR: Estimated glomerular filtration rate
ESC: European society of cardiology
ESS: Epworth sleepiness scale
FDA: U.S. Food and Drug Administration
GABA: Gamma-aminobutyric acid
GIP: Glucose-dependent insulinotropic polypeptide
GLP-1 RA: Glucagon-like peptide 1 receptor agonist
HbA1c: Glycated hemoglobin A1c
HCC: Hepatocellular carcinoma
HF: Heart failure
HFmrEF: Heart failure with mildly reduced ejection fraction
HFpEF: Heart failure with preserved ejection fraction
HFrEF: Heart failure with reduced ejection fraction
HHF: Hospitalization for heart failure
HR: Hazard ratio
hsCRP: High sensitivity CRP
IASP: International Society for Pediatric and Adolescent Diabetes
KCCQ-CSS: Kansas City Cardiomyopathy Questionnaire clinical summary score
KCCQ-OSS: Kansas City Cardiomyopathy Questionnaire overall symptom score
LV: Left ventricular
LVEF: Left ventricular ejection fraction
MACE: Major adverse cardiovascular event
MASLD: Metabolic dysfunction-associated steatotic liver disease
MASH: Metabolic dysfunction-associated steatohepatitis
MI: Myocardial infarction
MNP: Micro-and nanoplastics
MTD: Maximum tolerated dose
NAFLD: Nonalcoholic fatty liver disease
NASH: Nonalcoholic steatohepatitis
nsMRA: Nonsteroidal mineralocorticoid receptor antagonist
NSS: Neuropathy symptom score
NYHA: New York Heart Association
OSA: Obstructive sleep apnea
PAP: Positive airway pressure therapy
PCDE: Primary Care Diabetes Europe
PROs: Patient-reported outcomes
RAS: Renin-angiotensin system
RAAS: Renin–angiotensin–aldosterone system
RCT: Randomized controlled trial
SGLT2i: Sodium-glucose cotransporter-2 inhibitor
T1D: Type 1 diabetes
T2D: Type 2 diabetes
TIR: Time in range
UACR: Urine albumin-to-creatinine ratio
